# Health insurance education strategies for increasing the insured among older population – a quasi experimental study in rural Kenya

**Published:** 2012-05-13

**Authors:** Josephat Nyagero, Roslyn Gakure, Magaret Keraka

**Affiliations:** 1 Institute of Tropical Medicine and Infectious Diseases (ITROMID), of Jomo Kenyatta University of Agriculture and Technology, Nairobi, Kenya; 2 Jomo Kenyatta University of Agriculture and Technology, Nairobi Town Campus, Kenya; 3 Department of Public Health, Kenyatta University, Nairobi, Kenya

**Keywords:** Insured, uninsured, older population, health insurance, education, multi-strategies, Kenya

## Abstract

**Introduction:**

The older population in most developing countries are uninsured and lack access to health services. This study assessed the extent to which a multi-strategy health insurance education intervention would increase the number of insured among the older population in rural Kenya.

**Methods:**

The quasi-experimental study prospectively followed 1,104 unpaired older persons (60 years or more) in a 10-month health insurance education and enrolment intervention. The adjusted odds ratios computed at 95% confidence interval using a binary logistic regression tested the association between being insured and the multi-strategies.

**Results:**

At baseline, the lack of adequate knowledge on health insurance (52.9%) and high premiums (38.1%) were the main reasons for being uninsured. The insured older persons increased three-fold (from 7.7% to 23.8%) in the experimental site but remained almost unchanged (from 4.0% to 4.6%) in the control. The computed adjusted odds ratio for variables with significance (p < 0.05) show that the older people who obtained health insurance education through the chief's public meeting, an adult daughter, an adult son, a relative-sister/brother, an agent of the National Hospital Insurance Fund, and a health insurance beneficiary were 2.6, 4.2, 2.8, 2.3, 2.5 and 2.5 times respectively more likely to be insured. Access to health insurance education using a combination of 1-3 strategies and >3 strategies predisposed the older people 14.3 times and 52.2 times respectively to being insured.

**Conclusion:**

Health insurance education through multiple strategies and their intensity and frequency were pivotal in increasing being insured among the older population in rural Kenya.

## Introduction

The disease burden among the older population in developing countries remains a challenge. This premise is based on the finding that older persons aged 60 years or more are less likely to be insured, and are more vulnerable to ill health and financial problems, especially when they are uninsured [1–3]. One of the reasons for the high disease burden among the sub-population in Kenya is the low utilization of available health facilities [4]. Although offering a vivid explanation for the low utilization of health facilities is complex, the low health insurance coverage, estimated at 9.8% and 2% among the Kenyan general population and those aged 65 or more years respectively, offers a good starting point [5]. The discrepancy here therefore is that about 98% of the 1,440,847 Kenyans aged 65 years or more are uninsured. The proportion of the insured seems to remain static at the 2003 level of 9.1% despite ongoing efforts by the government and insurance companies to persuade Kenyans to register for health insurance [1, 5]. It is possible that the level of knowledge on the health insurance, socio-demographic and economic factors have a role to play in the low insurance levels as well as the high premiums charged to those in the older age category who are poor [1, 4, 6].

Whereas the younger population has opportunities to access a wide range of health insurance products, the older population and the retirees may only access the health insurance products provided by the National Hospital Insurance Fund (NHIF), a public sector agency [7]. Many employees discontinue with health insurance upon retirement. The retirees fail to remit their premiums for varied reasons, including; financial constraints, inaccessibility to previous health service providers upon returning to their rural homes, deliberate exclusion by insurance companies because of their age limit policies, or perceived expected support from adult children [6]. A limited number of tailor-made health insurance products from the private sector are accessible to the older population. Such products include the *Kinga ya Mkulima* (protection of the (tea) farmer) of Majani Insurance Brokers (MIB) and Senior Citizens Benefits Medical plan of Skylark African Insurance Broker affiliated to the British American Insurance and Madison Insurance companies respectively [8, 9].

Despite the achievement of significant improvement in the provision of health services by the Kenyan government and the private sector, health care access for the older population in Kenya remains a challenge [10]. The situation has become more complicated with the fast growth in numbers of the older population in the country, currently estimated at 4.2% per year [11]. The analysis of Household Health Expenditure and Utilisation Survey in Kenya showed that around 4% of households were facing catastrophic health expenditure [12]. Catastrophic expenditure is defined as out-of-pocket health expenditure (including routine health expenditures) representing 40% or more of total non-subsistence expenditure. The analysis also found that the lower income groups which include the older persons were more likely to face catastrophic health expenditure than higher income households [12]. Overall, 1.5% of Kenyan households were estimated to be impoverished due to catastrophic health expenditures [13].

The baseline results of this study showed that the most mentioned reason (52.2%) for being uninsured among the older population was lack of adequate knowledge on the benefits, sources and access procedures to being insured [14]. It was imperative to initiate a comprehensive and intensive health education and promotion programme intervention for increasing health insurance coverage among the older people in the study site. This intervention programme was designed based on both Rogers’ diffusion theory [15] and Lasswell's communication model [16]. In Rodger's theory, diffusion refers to the five-stage process by which an innovation is communicated through certain channels over time among the members of a social system till adoption. The innovation is defined as an idea, practice or object perceived as new by an individual or other unit of adoption [15]. The ownership of health insurance among the older population in the study area was the innovation. This study sought to assess the extent to which an intensive health insurance education intervention programme can increase health insurance coverage for the older population. This is in line with Lasswell's communication model which is analysed in five parts, S-M-C-R-E, i.e., sender-message-channel-receiver-effect [16]. In this study the sender are the health insurance providers, the message (new idea) is the information on health insurance, channels are the 18 different strategies of interpersonal or mass communication actions used in the 10-month intervention period, receivers are the targeted older population, and finally the effects is the older people's decision to enrol or not enrol for health insurance. According to Rogers, the S-M-C-R-E communication model corresponds to the elements of diffusion.

## Methods

The study utilized the quasi-experimental design [17–19] where two administrative locations in rural Kisii County were randomly selected and assigned as the experimental and control sites. The older persons aged 60 years or more were screened for health insurance ownership from both the public and/or the private sectors in the two sites. An unpaired design was preferred and a sample of 1,104 (559 from experiment site and 545 from the control site) was collectively tracked between the baseline and follow-up conducted 12 months later. As required in the unpaired design, a register of all the interviewed older persons with adequate personal contact details at baseline was made and used to trace interviewees during the follow-up. A consent form was signed independent of the interview schedule. In order to enhance anonymity, no unique identifier was used to either capture or enable the respondent's data to be linked back to them as required for the paired design [20]. Further, no new random sample was selected from the population during the follow-up as the list developed during baseline was used to identify the respondents [20].

**Figure 1:**
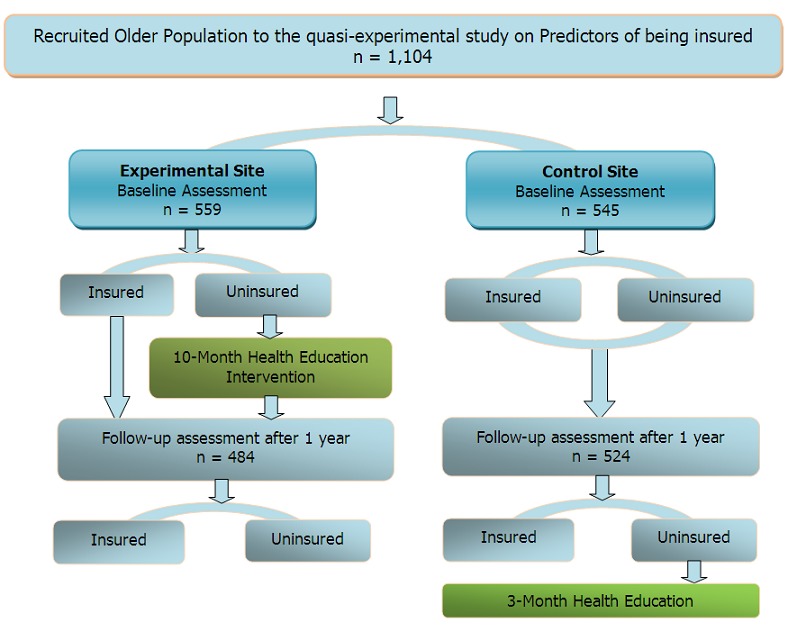
Flow diagram showing the older persons’ participation in the study

A 10-month intervention programme targeting the recruited cohort of uninsured older persons was implemented in the experimental study site. A follow-up in both the experimental and control sites was then carried out for comparison at the end of 10-month intervention. Based on the follow-up findings, a model on strategies of how best to increase access to health insurance cover amongst the older population in rural Kenya was empirically determined. For ethical reasons, the intervention activities were introduced in the control site soon after the completion of the follow-up study. Figure 1 summarizes the design for the study.

The design of the intervention started with a one-day consultative meeting which brought together leaders from all social structures within the experimental site. During the meeting the leaders were sensitized on the importance of encouraging the older persons to enrol for health insurance and the appropriate strategies to be used were agreed upon. The study endeavoured to increase the ownership of health insurance among the older population through intensive health education activities using a set of six-prong strategies. Separate activities were used to reach out to the older people with information on enrolment with a health insurance provider. The 6-prong strategies used included; the use of existing social/ community structures, family structures, health insurance agencies (mainly NHIF and Kenya Tea Development Authority-KTDA), study project staff, local FM radio and IEC materials. The data on these issues was collected during the follow-up only after completion of the 10-month implementation of the project's intervention activities. A comparison was then made between the findings obtained in the intervention and control sites. The frequency at which each of the respondents was reached either through field visits, interactions or participation in discussions and fora that encouraged enrolment with a health insurance provider was used as the indicator to measure the overall influence of the intervention. Table 1 shows the description and measurement of the intervention strategies adopted in the study.

**Table 1:** Description and measurement of the selected strategies used in the intervention

Strategy	Description/ measurement of intervention strategy
Chief/Elder's meeting	1 = if attended meeting and health insurance discussed; 0 = otherwise
Church	1 = if attended meeting and health insurance discussed; 0 = otherwise
Tea buying centre	1 = if at tea buying centre & health insurance discussed; 0 = otherwise
Funeral	1 = if attended a funeral and health insurance discussed; 0 = otherwise
Health insurance beneficiary	1 = if talked to by a health insurance beneficiary; 0 = otherwise
Community friend	1 = if talked to by a community friend; 0 = otherwise
Spouse	1 = if talked to by a spouse on health insurance; 0 = otherwise
Adult daughter	1 = if talked to by an adult daughter on health insurance; 0= otherwise
Adult son	1 = if talked to by an adult son on health insurance; 0 = otherwise
Relative	1 = if talked to by any other relative on HI; 0 = otherwise
NHIF representative	1 = if visited by an NHIF representative; 0 = otherwise
MIB representative	1 = if visited by an MIB representative; 0 = otherwise
Project staff	1 = if visited by a project staff; 0 = otherwise
Local FM announcement	1 = if listened to local FM announcement on HI; 0 = otherwise
Local FM road show	1 = if watched a local FM road show on HI; 0 = otherwise
Group meeting	1 = if attended a group meeting & HI discussed; 0 = otherwise
IEC materials	1 = if saw IEC materials on HI ownership; 0 = otherwise

### Statistical analysis

The univariate analytical procedures were utilized to initially manipulate the study variables. This paved way for the bivariate analysis and the Pearson's chi-square test was used to measure the association between the older population's participation in the selected strategies for health intervention and the study sites (experimental versus the control). The association between being insured and each of the selected intervention strategies was tested using Pearson's chi square test. The threshold for statistical significance was set at a = 0.05 and a two-sided p value at 95% confidence intervals captured for the corresponding analysis. All intervention strategies that exhibited statistical significance with being insured at bivariate level were included in the multivariate binary logistic regression model. In this analysis the backward conditional method was specified and performed in order to identify the confounders and effect modifiers. The resultant adjusted odds ratios were used to estimate the strength of association between the retained intervention strategies in the parsimonious model and being insured.

### Ethical issues

The research proposal was approved by the National Ethical Review Committee (NERC). Only the eligible persons who gave written informed consent were recruited, interviewed and followed up in the study. In addition, confidentiality was reassured during the interview and all the identifiers removed prior to data analysis and report writing. The respondents were also assured of their freedom to withdraw at any time during the study period.

## Results

Two surveys were carried out, first at baseline and second at follow-up upon completion of a 10-month intensive health education intervention. The surveys were held 12 months apart. The baseline solicited information from 1,104 older people recruited into the study. The follow-up survey interviewed 1,008 respondents in which 96(8.7%) of the respondents interviewed in the baseline failed to be included for various reasons. Fifty-six (56) of the baseline respondents had died within the 12-month period, representing an annual mortality rate of about 5.1% among the older persons. The other reasons for failure to be interviewed were that: the older people had moved to join their adult children residing outside the study sites (18, 19%), declined to consent for the follow-up interview (14, 15%), and being in hospital at the time of the follow-up survey (8, 8%). The subsequent results are therefore based on the 1,004 older persons who responded during the follow-up survey.

The health insurance coverage was measured in the baseline and follow-up survey by analyzing responses to two questions: Have you ever owned a health insurance cover in your lifetime? Are you currently on any health insurance cover? The proportion of the older population who had ever been insured increased from 12.9% to 18.3% during the inter survey period. Similarly, the proportion of the older population currently insured increased two-fold from 5.9% to 13.8% in the baseline and follow-up survey respectively. The vast majority were insured by the National Hospital Insurance Fund with 5.7% and 12.1% compared to the Majani Insurance Brokers’ 1.1% and 1.7% at baseline and follow-up, respectively. It is worth noting that whereas the currently insured older population almost remained constant in the control site during the baseline (4.0%) and follow-up (4.6%), it increased three-fold, from 7.7% at baseline to 23.8% during follow-up in the experimental site.

The health insurance education intervention strategy was identified based on the baseline survey results which showed that 52.9% of the older population lacked adequate knowledge on health insurance. Information on these strategies was therefore not collected during the baseline survey. The results presented in Table 2 show that the highest proportion (56.7%) of the older people in the intervention site were reached with health insurance information through the project staff. This was followed by a local FM radio presenter's road show, the churches, NHIF representatives, chief's/elder's public meeting (*baraza*: A baraza is the community meeting held periodically by the chief. It is used as the traditional method of informing villagers about various events and issues that are of importance to the community. Health insurance information was passed on to the older population through this forum.). The older person's adult son, with relatives and IEC materials were the least source of the information. For the control site, the highest proportion of the older people (10.1%) was reached with health insurance information through the tea buying centre followed by the adult son (9.7%) and a health insurance beneficiary (5.7%). The others were negligibly low (Table 2). Some of the older people had tea as a cash crop which they sold through the Kenya Tea Development Authority (KTDA) tea buying centre. The KTDA had representatives of Majani Insurance Brokers who periodically visited the tea buying centres to meet and encourage farmers to enrol for health insurance with MIB. The chi square test results (Table 2) shows that there were statistical differences in all the strategies used to reach the older people in the experimental and control sites with computed p values <0.05.

**Table 2:** Intervention activity old people interacted with that encouraged ownership of health insurance

Intervention activity that encouraged ownership of health insurance	Total n = 1008	Intervention	Control	P-value
		n = 484	n= 524	
**Social structures interventions**				
Chief/village elder's meeting	18	35.3	1.9	<0.001
Encouraged at church	21	42.8	1	<0.001
Encouraged by social group meeting	8.4	17.1	0.4	<0.001
Encouraged at tea buying centre	18.6	27.7	10.1	<0.001
Encouraged at a funeral function	13.3	24.8	2.7	<0.001
Encouraged by a community friend	7.6	12.8	2.9	<0.001
**Family based interventions**				
Encouraged by spouse	6.3	11.2	1.7	<0.001
Encouraged by adult daughter	10.6	18.6	3.2	<0.001
Encouraged by adult son	20.1	31.4	9.7	<0.001
Encouraged by a relative	4.2	5.8	2.7	0.013
**Health insurance agency**				
Encouraged by NHIF representative	20.4	40.9	1.5	<0.001
Encouraged by KTDA representative	8.4	14.7	2.7	<0.001
Encouraged by a health insurance beneficiary	8.8	12.2	5.7	<0.001
**Elderly project staff**				
Encouraged by elderly project representative	28	56.8	1.3	<0.001
Encouraged by elderly village representative	17	35.3	0	<0.001
**Local FM radio interventions**				
Encouraged by local FM announcement	17.3	34.9	1	<0.001
Encouraged by local FM road show	25.5	51	0	<0.001
**IEC materials**				
Encouraged through IEC materials	2.9	6	0	<0.001

The intensity of the health insurance education was measured using the number of the different strategies/frequency with which the older people were reached with the health insurance education. At the experimental site, the mean number of strategies/times the older people had been reached with health insurance education was 4.79 (SD±3.15, median 5, range 0-18) compared to a mean of 0.49 (SD±0.927, median 0.00, range 0-9) in the control site.

Bivariate analysis results presented in Table 3 shows that the health education activities implemented during the intervention phase were significantly associated with being insured at follow-up with all having p-values of <0.001 and appropriate odds ratios. However, upon adjustment for confounders, the adjusted odds ratios (AOR) for six of the 18 intervention activities were significant. Being encouraged to take up health insurance cover at a chief's/village elder's public meeting predisposed the older people 2.2 times to enrolment for health insurance (AOR = 2.2; 95% CI 1.3-3.9; p = 0.004). The use of a health insurance beneficiary to reach the older people predisposed them 2.4 times to enrolment for a health insurance (AOR = 2.4; 95% CI 1.3-4.4; p = 0.006). The adult daughter and adult son as a key study intervention predisposed the older people to enrolment for health insurance 3.7 times (AOR = 3.7; 95% CI 2.1-6.3; p < 0.001), and 2.6 times (AOR = 2.6; 95% CI 1.6-4.2; p < 0.001) respectively. Through the project, a National Hospital Insurance Fund representative periodically visited the older people with health insurance information and provision of the eventual enrolment services. Those reached by the NHIF representative were 2.3 times more likely to enrol for a health insurance (AOR = 2.3; 95% CI 1.4-4.0; p < 0.002). Contrary to expectations, the older people who listened to the appeal made through the local FM radio station to enrol for health insurance were 60.0% less likely to enrol for a health insurance compared to those not listening to the appeal (AOR = 0.4; 95% CI: 0.2-0.8; p < 0.005). The analysis of being insured in relation to the number of intervention strategies used to encourage the respondent to enrol for health insurance found that being reached with 1-3 intervention strategies predisposed the older people 14.3 times to being insured (AOR = 14.3; 95% CI 5.0-41.1; p < 0.001) compared to no strategy. Further, being reached by >3 intervention strategies predisposed the older people 52.2 times to enrolment for health insurance (AOR = 52.2; 95% CI 19.0-143.4; p < 0.001) compared to not being reached by any strategy. At multivariate level, a binary logistic regression was used to model health insurance coverage (0 = uninsured, 1 = insured) using all the 18 intervention actions listed in Table 3. These intervention actions were significantly associated (independently) with being insured among the older population at bivariate analysis. After successive iterations was performed using backward conditional method in order to identify confounders and/or effect modifiers, eight intervention strategies were identified as being associated with health insurance enrolment among the older population. The resulting parsimonious model is shown in Table 4 and captures the adjusted odds ratio (AOR) with their respective 95% confidence interval and p value for each of the intervention action significantly associated with the older people owning a health insurance.

**Table 3:** Relationship between health insurance coverage and health education activities

Encouraged at:	Owns HI (n = 139)		Doesn't own HI (n = 869)		Bivariate analysis				Multivariate analysis			
					OR	95% CI of OR		P value	AOR	95% CI of AOR		P value
	N	%	n	%		Lower	Upper			Lower	Upper	
Chief/Elder's meeting*	59	32.6	122	67.4	4.5	3.1	6.6	<0.001	2.2	1.3	3.9	0.004ө
Church*	67	31.6	145	68.4	4.6	3.2	6.8	<0.001	1.5	0.9	2.4	0.145
Tea Buying Centre*	40	21.4	147	78.6	2	1.3	3	0.001	0.9	0.5	1.5	0.575
Funeral*	37	27.6	97	72.4	2.9	1.9	4.4	<0.001	0.8	0.5	1.5	0.549
HI Beneficiary*	32	36	57	64	4.3	2.6	6.9	<0.001	2.4	1.3	4.4	0.006 ө
Community friend*	24	31.2	53	68.8	3.2	1.9	5.4	<0.001	1.1	0.6	2.2	0.766
Spouse*	27	42.9	36	57.1	5.6	3.3	9.5	<0.001	1.7	0.8	3.5	0.15
Adult daughter*	51	47.7	56	52.3	8.4	5.4	13	<0.001	3.7	2.1	6.3	<0.001 ө
Adult son*	69	34	134	66	5.4	3.7	7.9	<0.001	2.6	1.6	4.2	<0.001 ө
Relative*	17	40.5	25	59.5	4.7	2.5	9	<0.001	2.2	1	5.1	0.059
NHIF Representative*	71	34.5	135	65.5	5.7	3.9	8.3	<0.001	2.3	1.4	4	0.002 ө
KTDA Representative*	21	24.7	64	75.3	2.2	1.3	3.8	0.002	1.2	0.6	2.3	0.663
Project Staff*	76	27	206	73	3.9	2.7	5.6	<0.001	1.1	0.6	2.1	0.664
Project Village staff*	50	29.2	121	70.8	3.5	2.3	5.2	<0.001	1.4	0.8	2.5	0.272
FM announcements*	38	21.8	136	78.2	2	1.3	3.1	0.001	0.4	0.2	0.8	0.005 ө
Local FM road show*	71	28.2	181	71.8	4	2.7	5.7	<0.001	1.5	0.8	2.8	0.198
Group meeting*	25	29.4	60	70.6	3	1.8	4.9	<0.001	1	0.5	1.9	0.946
IEC Material*	9	31	20	69	2.9	1.3	6.6	0.006	0.7	0.3	2	0.535

HI, Health insurance

* Reference category used, No

ө Significant at 0.05 level; NHIF: National Hospital Insurance Fund; KTDA: Kenya Tea Development Authorit

**Table 4:** Logistic regression model predicting health insurance coverage based on the intervention actions

Variables	AOR	95% CI of AOR		P value
		Lower	Upper	
**Social structure interventions**				
Encouraged in a chief/elder's meeting* = Yes	2.6	1.5	4.3	<0.001ө
**Family based interventions**				
Encouraged by adult daughter* = Yes	4.2	2.5	7	<0.001ө
Encouraged by adult son* = Yes	2.8	1.8	4.4	<0.001ө
Encouraged by relative* = Yes	2.3	1	5.3	0.043ө
**Health insurance agency**				
Encouraged by NHIF Representative* = Yes	2.5	1.5	4.3	<0.001ө
Encouraged by HI beneficiary* = Yes	2.5	1.4	4.5	0.002ө
**Local radio interventions**				
Encouraged by local FM announcement* = Yes	0.4	0.2	0.7	0.003ө
Encouraged by local FM Road show* = Yes	1.8	1.1	3	0.026ө

Reference category used, **No**

ө Significant at 0.05 level; NHIF: National Hospital Insurance Fund

Adjusting for all the other intervention actions, the older people who attended the chief's/ elder's public meeting were 2.6 times more likely to enrol for health insurance compared to those who did not attend (AOR = 2.6; 95% CI 1.5-4.3; p)

Adjusting for all the other intervention actions, use of the adult daughter, adult son and a relative separately as family-based interventions to encourage the older people to enrol for health insurance were significantly associated with being insured. The older people encouraged by their adult daughter, adult son and a relative were 4.2 times, 2.8 times and 2.3 times more likely to enrol for health insurance compared to those not encouraged by respective counterparts. However, being encouraged by the spouse as a family-related intervention action was not found to be associated with being insured.

When adjustment was done for other intervention actions, use of a National Hospital Insurance Fund (NHIF) representative and use of a health insurance beneficiary separately as health insurance agency-based strategies to encourage the older people to enrol for health insurance were significantly associated with being insured. The older people who were encouraged by an agent from the National Hospital Insurance Fund and a health insurance beneficiary were both 2.5 times more likely to enrol for health insurance compared to those not encouraged by the agent or the beneficiary. However, being encouraged by the agent from the Kenya Development Tea Authority (KTDA) was not associated with being insured.

Adjusting for other intervention actions, use of the local FM radio announcements programme and the local FM radio road show were separately found to be significantly associated with being insured. The older people who were encouraged by an announcement in the local FM radio in the local language were 60.0% less likely to enrol for health insurance compared to those who did not listen to the announcement (AOR = 0.4; 95% CI: 0.2-0.7; p = 0.003). However, the older people who witnessed the road show involving one popular local FM radio presenter and one comedian in the same station were 1.8 times more likely to enrol for a health insurance compared to those who did not witness the road show done in the intervention site.

It is worth noting that the intervention actions for the project staff and those related to information, education and communication materials (IEC) were not found to be significantly associated with enrolment of health insurance among the old people in the study area.

## Discussion

The use of a quasi-experimental design offered an opportunity to implement interventional activities in the experimental site and compare empirical results to those of the control site in both the baseline and follow-up survey. It was through such a design that the differences in the effectiveness of strategies used to carry out the health insurance education and enrolment of the older population into insurance could be best measured [17–19]. Although the study did not use the higher level paired design to track respondents in the baseline and follow-up, the adopted unpaired design using the register of interviewed older persons at baseline made it possible to effectively enable collective tracking of respondents between the two surveys [20].

The reported loss to follow up of respondents was relatively high. However, the reasons presented for this loss were prudent. For instance, the reported mortality level within a period of 12 months is in conformity with the mortality patterns for the older population. The State of Kenya Population Status report indicated that the life expectancy for males at age 55 was projected at 18.4 years while that of females was 24.0 years [21]. This implies that the observed higher death rate among the older population aged 60 years or more was in conformity with the report on the State of Kenya Population 2011. The other reasons for failure to be interviewed in the follow-up confirms that Kenya is still based on family systems with collective society structures because the adult children continue to have an obligation to support the older persons [22]. This is because a significant proportion of the enlisted older persons would not be interviewed at follow-up as they had moved outside of the study area to stay with their adult children. Equally, having a substantial number of the older population not interviewed at follow-up for being in hospital is evidence that the sub-population is prone to ill health, partly because of their age. Similar results have been reported for example, among a sample of 320 Indian older population, the average illness per person was 2.77 [23].

The proportion of currently insured older people at baseline was within the 2-8.4% range previously reported in the same sub-population [24]. However, the proportion of currently insured older persons at follow-up was more than double the 9.1-9.8% for the general population [24, 25]. The present study results confirm that the public sector agency, the National Hospital Insurance Fund, remains the main source of health insurance in developing countries [24, 25]. After adjustment, the baseline results demonstrated equivalence in ownership of health insurance between the control and the experimental (no significant difference). At follow-up, there were significant differences in the ownership of health insurance between the control and the experimental site, implying that the health insurance education intervention worked out successfully. Indeed, the proportion of the insured older persons at follow-up was a more than three-fold increase in the experimental site compared to the national level for the general population [14, 24]. The achievement of such a high proportion can substantially be attributed to the impact of the 10-month health insurance education intervention activities implemented in the intervention site.

Whereas the observed increase in the proportion of the insured older persons in the experimental site is appreciated, it still remains low for accelerated universal access to quality and specialised health care services among the older population in rural Kenya. For example, by 2010, the USA government planned to allocate more than 8 percent of the government's GDP to expenditure on Social Security and Medicare 2010 [26]. Such schemes are not existent in Kenya yet; and this may symbolize how far the country is from creating an enabling environment for attaining universal access to health care services to its senior citizenry.

The study's intervention programme and activities was a product of a participatory process which was informed by baseline findings as required in quasi experimental study designs [19]. The key baseline findings were presented in a consultative meeting held with the community leaders and other health insurance stakeholders. The output of the consultative meeting was a set of 18 actions for implementation in the health education programme. Such a participatory process enhanced the ownership and motivation to implement the programme activities during the 10-month intervention life circle.

Using the chief's public meetings (*baraza*) to encourage the older population to enrol for health insurance confirmed this commonly used approach as a social structural strategy that is effective in the adoption of new ideas or innovation in a rural setting. The public meetings have previously been effectively used in the introduction and adoption of other development related actions [27]. The separate use of the adult daughter, adult son and a relative as family-based intervention strategy actions to encourage the older people to enrol for a health insurance confirmed findings of previous studies [22, 28, 29]. The use of health insurance agents and beneficiaries to convince potential older persons to enrol for health insurance was equally confirmed. It is therefore not surprising that a majority of insurance agencies use both the agents and beneficiaries to reach out to potential clients in the marketing of their products. The use and power of the media to encourage the adoption of new ideas was in a rural setting was also similar to previous studies. For example, a study in India found that the radio, television, print, and internet were pivotal in influencing the symbolic adoption behavior of rural women on rabbit farming technologies [30]. However, in the present study, IEC print materials did not encourage the enrolment among the older population, probably because of the literacy levels and other setbacks associated with old age.

## Conclusion

Use of existing social structures, family members, health insurance agency representatives and local radio stations are effective strategies that can be used to encourage the older population to be insured. The specific effective actions in encouraging the older population to enrol for health insurance are the use of the chief/elder's meeting or *barazas*, encouragement by adult daughter, son or relative, use of health insurance representatives, health insurance beneficiary, placement of announcements in the local radio stations, and organising road shows. Concerted effort should be made to increase the level of knowledge about health insurance among the older population in the rural setting using participatory identified strategies.
